# Wearable Device to Monitor Back Movements Using an Inductive Textile Sensor

**DOI:** 10.3390/s20030905

**Published:** 2020-02-08

**Authors:** Astrid García Patiño, Mahta Khoshnam, Carlo Menon

**Affiliations:** Menrva Research Group, Schools of Mechatronic Systems & Engineering Science, Simon Fraser University, Metro Vancouver, BC V5A1S6, Canada; agarciap@sfu.ca (A.G.P.);

**Keywords:** inductance, nurses, low back pain, textile sensors, trunk posture, monitoring, wearable device, smart garment, E-textiles

## Abstract

Low back pain (LBP) is the most common work-related musculoskeletal disorder among healthcare workers and is directly related to long hours of working in twisted/bent postures or with awkward trunk movements. It has already been established that providing relevant feedback helps individuals to maintain better body posture during the activities of daily living. With the goal of preventing LBP through objective monitoring of back posture, this paper proposes a wireless, comfortable, and compact textile-based wearable platform to track trunk movements when the user bends forward. The smart garment developed for this purpose was prototyped with an inductive sensor formed by sewing a copper wire into an elastic fabric in a zigzag pattern. The results of an extensive simulation study showed that this unique design increases the inductance value of the sensor, and, consequently, improves its resolution. Furthermore, experimental evaluation on a healthy participant confirmed that the proposed wearable system with the suggested sensor design can easily detect forward bending movements. The evaluation scenario was then extended to also include twisting and lateral bending of the trunk, and it was observed that the proposed design can successfully discriminate such movements from forward bending of the trunk. Results of the magnetic interference test showed that, most notably, moving a cellphone towards the unworn prototype affects sensor readings, however, manipulating a cellphone, when wearing the prototype, did not affect the capability of the sensor in detecting forward bends. The proposed platform is a promising step toward developing wearable systems to monitor back posture in order to prevent or treat LBP.

## 1. Introduction

Working for long hours in occupations such as nursing careers, that involve frequent awkward trunk movements and repetitive twisting or bending of the trunk, increases the risk of low back pain (LBP) [1,2,3,4]. Considering that LBP is the most common work-related musculoskeletal disorder [1,2,3], it is important to establish guidelines toward its prevention and treatment. An effective step in this regard is to educate individuals about their body posture during the activities of daily living and throughout the therapeutic protocols that are prescribed for prevention or treatment of LBP [5,6,7]. 

Considering the relationship between the body posture and LBP, it could be beneficial to monitor back movements and provide relevant feedback to the individuals. Previous studies have collected such information using questionnaires [2,3,5,6,7,8], accelerometers [1,9,10], and other types of technologies such as optical motion-tracking systems [11,12,13]. However, these solutions have limited practicality since questionnaire results might be subjective, data collected with accelerometers might be inconsistent due to sliding or removal of sensors, and cameras and similar motion-capture systems are bulky with long setup times that can be accommodated mostly in dedicated clinical environments.

Electronic textiles (E-textiles) or smart garments provide a viable wearable solution for developing standalone platforms that can objectively monitor back movements. In such systems, electronic components and/or textile sensors such as inertial measurement units (IMUs), capacitive, resistive or inductive sensors, and light-emission diodes (LEDs) are embedded within the fabric [14,15,16,17]. Some of the applications of such platforms in healthcare are electrocardiography (ECG), sports research, plethysmography, postural monitoring, movement analysis, and muscle activity measurements [14,17,18]. E-textiles have also been considered in developing wearable and comfortable movement tracking platforms due to their small size, lightweight, and simple operation that allow for unobtrusive monitoring of user movements during activities of daily living [15,19]. 

Resistive textile sensors are made in a variety of shapes with various production techniques, such as incorporating conductive threads (e.g., carbon nanotubes, silver-coated thread, copper wire) into the textile which can be done by sewing, embroidery, weaving, knitting or braiding machines. Coating non-conductive threads (e.g., metals, galvanic substances, metallic salts) with conductive material and printing conductive ink into the textile are other production techniques [15,16]. The fundamental working principle of resistive sensors is that any mechanical deformation of the sensor results in a change in its electrical resistance [18,19,20]. Resistive textile sensors are characterized with high hysteresis, non-linearity of their response, and a drift in their readings when a certain amount of stretch is held for a period of time [15]. 

Capacitive textile sensors are formed by two or more conductive plates and a dielectric element. Conductive plates can be fabricated by different methods, such as sewing, embroidering or weaving conductive material (e.g., conductive threads) into the fabric and coating or painting a section of the textile with conductive ink [16]. Another method is to use conductive polymers as the conductive plates to be attached to the textile [15]. The dielectric component required between the conductive plates can be made using soft non-conductive polymers, foams, or fabric spacers [16]. The fundamental working principle of capacitive sensors is that the capacitance value depends on the distance between the conductive plates. In other words, when conductive plates become closer to each other as a result of applied pressure, the capacitance value increases. In comparison with resistive textile sensors, these sensors demonstrate more linear behavior, less hysteresis, and faster response [15,16]. However, the manufacturing process of capacitive sensors is more complicated and requires more equipment than the resistive sensors [15].

Inductive textile sensors are made from highly conductive materials, such as copper wire, stainless steel yarn, or conductive threads that combine different alloys. These sensors typically have a loop configuration with a circular geometry, although they might also be manufactured in other shapes such as square, rectangular, and pentagon [21]. The possibility of manufacturing inductive sensors in various shapes grants them the versatility to be embedded in or affixed to different surfaces. Consequently, inductive sensors are regularly used in antennas [22,23] and plethysmographs [21,22,24]. Furthermore, this type of sensors has also been used in wireless-powered applications [25], data transmission [25], monitoring heart rate [26], and measuring strain and displacement [27].

In healthcare, resistive textile sensors have been successfully used in wearable platforms for back posture monitoring [19,28,29]. However, the aforementioned disadvantages of resistive textile sensors limit their practicality [29]. In this paper, we introduce a wireless inductive textile sensor-based wearable device to monitor trunk movements. This study is a preliminary study towards developing a lightweight and comfortable wearable device with long battery life which is well-suited for objective monitoring of forward bending of trunk during longer periods of time.

## 2. Sensor Design and Validation through Simulation

Inductive textile sensors are considered as the sensing modality for this study. The working principle of these sensors is that when electrical current passes through loop(s) of conductive threads, a magnetic field is created. Sensor deformation due to externally applied force affects the shape of the magnetic field and, thus, changes the sensor output. Consequently, it is possible to increase the inductance and sensitivity (Δ Inductance/Δ strain) of the sensor through augmenting the number of coils and/or narrowing the width and space between the coils [30].

### 2.1. Configuration of the Inductive Textile Sensor 

The focus of this study is to detect forward bending of the trunk and discriminate it from other movements such as lateral bending or twisting. To achieve this goal, the configuration and placement of the sensor should be chosen strategically. Previous studies reported that when an individual wearing a tight-fitting shirt bends forward, the lumbar section of the back undergoes major strain [29]. However, trunk movements in frontal and horizontal planes, which correspond to lateral bending and rotation, cause a smaller strain on this section [29]. According to such evidence, the inductive sensor in this study was created by arranging a copper wire in a zigzag pattern to form an inverted “T” shape. The idea here was to position the horizontal part of the inverted “T” on the lumbar section of the back to capture strain variations due to trunk movements and use the vertical part as a framework to align the shirt with the spine. Apart from helping with alignment, the vertical part of the sensor had practical implications: placing the circuitry on the upper area of the back is more practical since the circuitry box would cause less discomfort to the user. Furthermore, continuing the connection of the sensor up to the circuitry avoids loose cables.

Sewing the sensor into the elastic fabric in a zigzag pattern allows the fabric to stretch without causing damage to or breaking the sensor. This is an important feature which improves the reliability of the system. Moreover, considering that the length of the wire affects the resulting electrical inductance, the proposed zigzag pattern increases inductance and, thus, improves sensor sensitivity as well as its resistance to interference from other electrical devices [30]. Furthermore, dimensions of the zigzag pattern, more specifically its length, angle, and width, are also determining factors in the resulting inductance [31]. 

### 2.2. Zigzag Pattern 

In this study, the length and the angle of the zigzag pattern should be chosen such that the fabric could be sufficiently stretched to accommodate full forward bending without breaking the sensor and the resulting inductance value. To achieve this, a series of Ansys simulations were performed to calculate the change on the inductance value based only on the width of the zigzag. Subsequently, three inductive sensors with higher simulated inductance value were selected for the next step which included an experimental evaluation. In this scenario, each sensor was manually stretched up to 200% of its original length in order to assess its resistance against rupture. The physical inductive sensors were made as similar as possible to the simulations.

**Figure 1:**
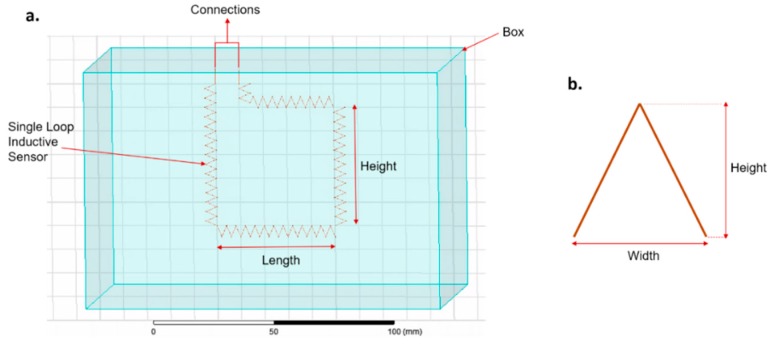
Zigzag pattern evaluation in Ansys. (**a**) Single loop inductive textile sensor; (**b**) definition of zigzag characteristics.

The parameters used in the Ansys simulations and the zigzag parameters are reported in Table 1. Figure 1a illustrates the characteristics of a single loop inductive sensor and corresponding Ansys parameters. All parameters of the Ansys simulations were kept constant except for the zigzag width. Figure 1b illustrates how the zigzag characteristics were defined.

**Figure 2:**
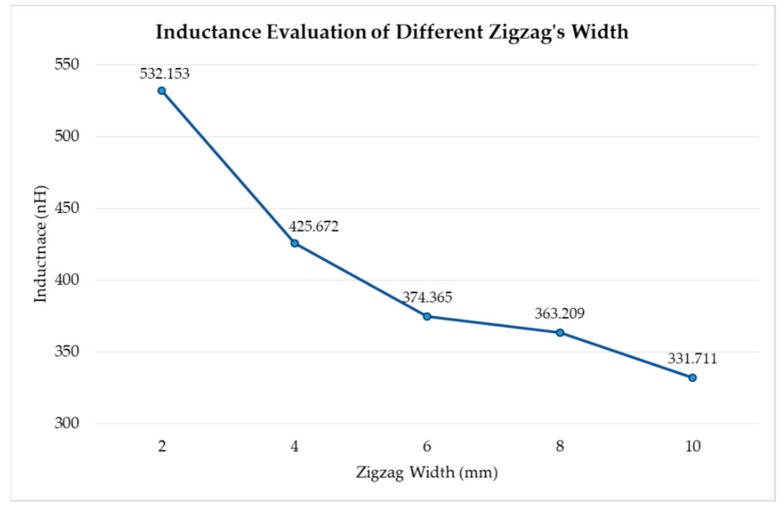
Inductance vs. zigzag width. Inductance values simulated in Ansys for a single-loop inductive sensor with changing the zigzag width.

The resulting inductance values of the 5 single loop sensors are shown in Figure 2. It is observed that for the same height, the inductance increases when the width of the zigzag decreases. The highest inductance value achieved with a single loop configuration was 532.153 nH at a width of 2 mm, while the lowest value of inductance was 331.711 nH achieved with a zigzag width of 10 mm.

In the next step, three single-loop inductive sensors with a zigzag width of 2, 4, and 6 mm corresponding to highest obtained inductance values (532.153, 425.672, and 347.365 nH, respectively) were constructed. Each inductive sensor was manually stretched up to 200% of its original length. The inductive sensors with zigzag width of 2 and 4 mm were successfully stretched without breaking; however, the sensor with the 6 mm zigzag width broke during the stretch.

According to the obtained inductance values, in terms of functionality, sensors with 2 and 4 mm of zigzag width could have a good performance. However, in terms of comfortability, having a smaller zigzag width increases the stiffness of the fabric and its weight, which could potentially interfere with the comfort of the user. Therefore, the zigzag with a 4 mm width was chosen due to its inductance high value and its resistibility to be stretched up to 200% of its original length.

### 2.3. Simulation Study 

A simulation study was performed in Ansys 17.2 Electromagnetics Suite (Ansys Inc., Canonsburg, PA) to evaluate the design concept proposed in the previous section. In this regard, the effect of the zigzag pattern on electrical inductance and the behavior of the magnetic field of the sensor were investigated. It should be noted that although trunk movements stretch the fabric in all three dimensions, in the simulation phase, to avoid overload of computer memory, only two-dimensional (2D) stretches were considered to reduce the complexity of equations and the computational time. 

**Figure 3:**
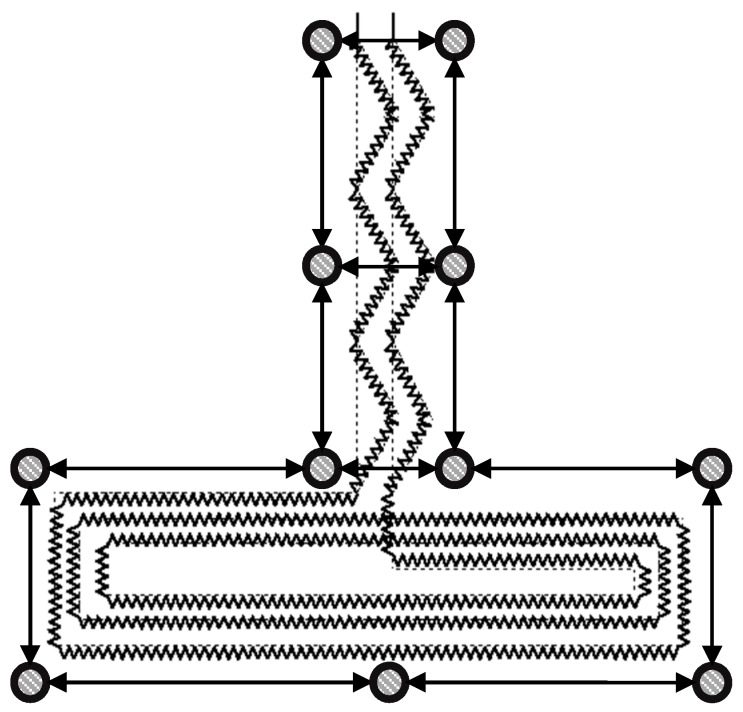
Placement of optical markers around the proposed shape for the inductive sensor. Markers are shown as grey circles.

To ensure that the parameters used in simulations were close to corresponding actual values, 11 reflective markers, with a diameter of 8 mm, were affixed to the selected piece of garment (a tight-fitting leotard as explained in Section 3.1) around the section designated for sewing the sensor, as shown in Figure 3. A participant was instructed to wear the garment, stand in an upright position, and then bend forward without flexing the knees. The position of optical markers during this move was recorded using a Vicon Motion Capture system (Vicon, Oxford, UK). Collected data were then analyzed in MATLAB R2017b (The MathWorks, Inc., Natick, MA) to measure the distance between each two reflective markers. Calculated distance values were used in the simulation to represent the dimension of sensors in their original as well as stretched condition. During this test, in the full forward bending position, the garment fabric was stretched to 104% and 132% of its original length in the horizontal and vertical direction, respectively. Therefore, the same stretch value was used in the simulation study. 

**Figure 4:**
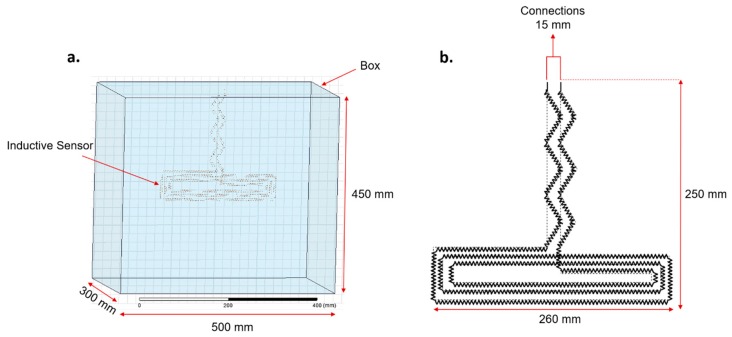
Ansys simulation of the inductive sensor: dimensions of the (**a**) box, (**b**) inductive sensor.

To facilitate changing parameter values during the simulation, such as sweep definitions in optometrics, the sensor geometry was built in Ansys Workstation V2.0, and its behavior was simulated in Ansys Maxwell 3D design. The parameters used to simulate the resulting inductive sensor are shown in Table 2. Figure 4 illustrates the dimensions of the box and the inductive sensor used in Ansys simulations. 

In Table 2, “Sensor characteristics” correspond to properties of the inductive textile sensor embedded on the textile. The parameter “Between connections” defines the distance between the two ends of the inductive sensor. For Ansys Maxwell 3D to run a simulation, it is necessary to delimit the space, denoted by “Box” in Table 2, and to specify the material of the object which in this case was air. The “Setup” parameters are [32]:

1. Maximum Number of Passes” defines a limit on the adaptively refined passes that the solver performs.
2. Percentage of Error” defines the goal for the Error Energy and Delta Energy.
3. Percentage of Refinement Per Pass” determines the number of tetrahedral elements added in the mesh refinement.
4. Minimum Number of Passes” defines the minimum number of adaptive passes before the simulation stops.
5. Minimum Converged Passes” determines the minimum number of adaptive passes that converged before the solution stops.

Moreover, “Mesh” is the computer process of redefining an object in finite number of tetrahedra, and “Excitation” is the current that runs through the sensor.

Simulation results showed that with the proposed sensor configuration, the inductance would increase from 4.698 µH in unstretched condition to 5.11 µH in maximum stretch, which is equivalent to an 8.8% increase in the inductance value. 

To investigate the effect of zigzag pattern, the simulation was repeated with considering an unstretched sensor without the zigzag pattern. Results indicated that the inductance value in this case would be 3.476 µH. Comparing this value with that obtained for the unstretched sensor with zigzag pattern, i.e., 4.698 µH, indicates that the zigzag pattern could increase the inductance value by 35%, which points to the effectiveness of the proposed configuration in increasing the sensitivity of the sensor.

**Figure 5:**
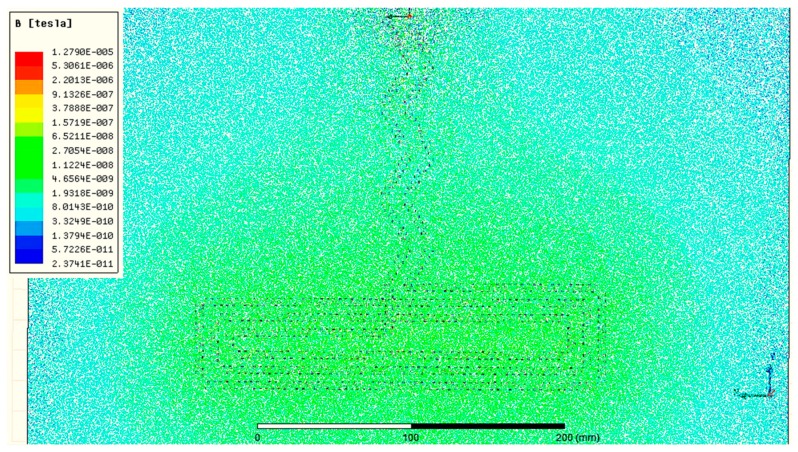
Simulation of the electromagnetic field created by the sensor.

To observe the electromagnetic field created by the sensor with the proposed geometry and configuration, another simulation study was undertaken using parameters in Table 2. The resulting simulated Magnetic Field B (tesla) is shown in Figure 5, where the red color represents the highest value and blue represents the lowest value. It is observed that with the proposed sensor design, the magnetic field is stronger around the horizontal section of the inverted “T”, which would be placed on the lumbar section of the back. This area of higher magnetic field is where the sensor is more sensitive, i.e., a small strain could noticeably change the inductance value. Therefore, such a sensor is well-suited for monitoring forward bending of the trunk (Section 2.1).

## 3. Sensor Prototype and Evaluation Protocol

### 3.1. Smart Garment Prototype

To develop the wearable back monitoring platform for this study, the inductive textile sensor prototype was integrated within a leotard, chosen for its comfortability, tightness, and stretching properties. Such a garment can be comfortably worn under a uniform, thus allowing the user to move freely without interfering with their performance of activities of daily living. 

**Figure 6:**
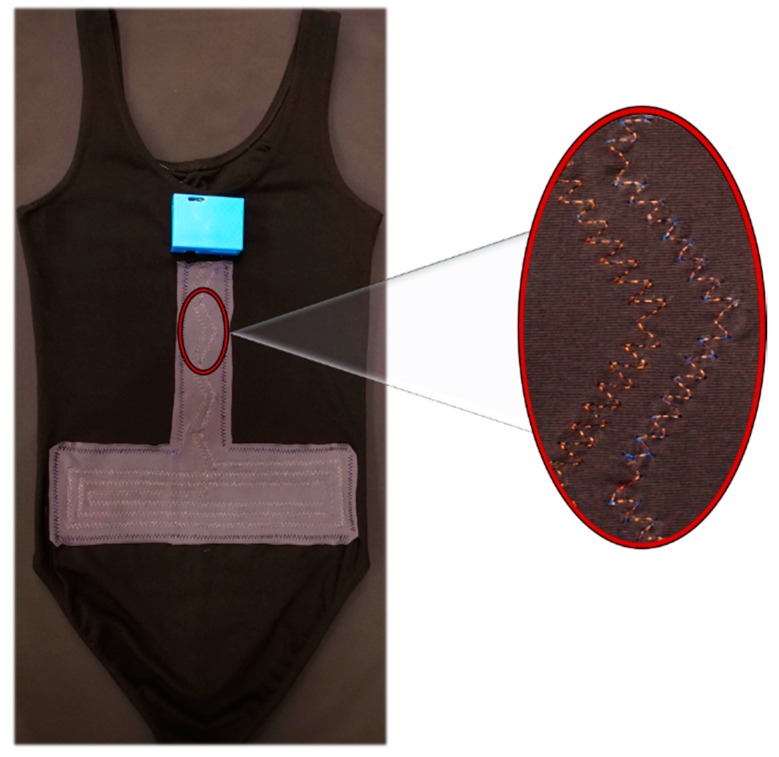
Smart garment prototype. Rear view of the smart garment with the inductive sensor affixed to the part that goes on the lumbar section.

To form the inductive sensor, a single copper wire with a diameter of 0.14 mm was sewn into a piece of elastic fabric in the discussed inverted “T” shape in a zigzag pattern (Section 2.1). The elastic fabric with the embedded inductive sensor was then affixed to the back of the leotard such that the vertical part of the inverted “T” was aligned with the spine and the horizontal part was placed on top of the lumbar section of the back as shown in Figure 6. The horizontal section of the inverted “T” was a flat coil with 3 concentric loops in a rectangular shape which were separated from each other by 1 cm. During the fabrication process, the inductance values were measured with a pair of smart tweezers (LCR Pro1, LCR Research, Toronto, ON, Canada) at a frequency of 100 Hz.

Wireless communication circuitry was designed to acquire sensor readings. More specifically, a high-resolution inductance-to-digital converter board (LDC1614, Texas Instruments Inc., Dallas, TX) collected inductance values from the sensor and transferred them to a microprocessor (Arduino Mini, ATMega328, Microchip Technology, Chandler, AZ) via I2C protocol. The microcontroller then communicated with a Bluetooth module (HC-06 Bluetooth Module, Guangzhou HC Information Technology Co., Ltd., Guangzhou, China) to transmit the received data to the user’s cellphone. A smartphone application was also developed to collect such data and store it on the phone for later processing. The inductance-to-digital board was powered with a LiPo battery providing 3.7 V and 1200 mAh, and the sampling rate of the prototype was 200 Hz.

### 3.2. Testing Protocol

To evaluate the performance of the sensor, one healthy participant (female, 25 years old, 161 cm) was asked to wear the instrumented garment and perform three cycles of the following movements:

1. Six repetitions of bending forward, as much as possible and comfortable, at a selected speed without bending the knees;
2. Three repetitions of bending to the right, standing straight, bending forward, standing straight, and then bending to the left;
3. Three repetitions of rotating the trunk to the right, standing straight, bending forward, standing straight, and then rotating the trunk to the left.

During these movements, the participant was asked to keep her hip as still as possible. To determine true forward bending angles (roll), two IMUs (Xsens Awinda, Enschede, Netherlands) were positioned on C7 and L5. The ethics of this study was approved by the Office of Research Ethics at Simon Fraser University, and the participant gave informed consent for her participation.

### 3.3. Interference Test

To investigate how the performance of the fabricated inductive sensor changes in proximity of other objects, such as magnet, metallic objects, or wireless devices, a two-phase interference test was designed:

1. In the first phase, the inductance value of the sensor was observed before and after different objects that could potentially interfere with sensor readings were brought close to the unworn garment. The chosen objects included: a copper spool (same material used for the inductive sensor with a length of 5.5 cm and a diameter of 2 cm), a disc-shaped metallic object (an alloy of iron, width = 1 cm, diameter = 3.7 cm), a disc-shaped magnet (width = 0.3 cm, diameter = 2.5 cm), a cellphone (device turned on with Wi-Fi activated), and a human hand. The prototype was fully extended on a table with the inductive sensor facing upward. The object was moved towards the inductive sensor from a distance to the proximity of the coil in vertical direction while the largest face of the objects was facing the coil. The objects were held in the proximity of the inductive sensor for approximately 8 s.
2. In the second phase, the participant was asked to wear the prototype and perform three cycles of the following protocol:
  (1) Stand upright without moving for approximately 15 s;
  (2) Five repetitions of forward bend, as much as possible without bending the knees, at a comfortable speed;
  (3) Pick up the phone from the table in front and put it inside the jeans’ back pocket;
  (4) Stand upright without moving for approximately 25 s;
  (5) Five repetitions of forward bend, as much as possible without bending the knees, at a comfortable speed.

### 3.4. Outcome Measures

In evaluating the performance of the prototype fabricated with the proposed sensor design, two main outcome measures were considered:

1. Current consumption, which is an indicative of the battery life of the sensing unit. A lower current consumption allows for monitoring back movements during longer periods of time, e.g., an entire work shift,
2. Inductance value, which is the electrical response of the sensor to externally applied strains. When the user bends forward, the sensors are stretched, resulting in higher inductance values.

## 4. Experimental Results

### 4.1. Current Consumption

Experimental tests showed that the total power consumption of the prototype was 20.1 mA. Therefore, the prototype can operate and transmit data for more than 10 h continuously.

### 4.2. Inductance Value

The inductance value of the sensor sewn on leotard before being worn was 4.5 µH, as measured by the smart tweezers. However, the inductance value reported by the converter board was 4.6 µH, which shows a difference of 2.22% between the two measurements. This comparison was performed mainly to ensure that the readings from the developed circuitry were reliable and representative of actual inductance values.

**Figure 7:**
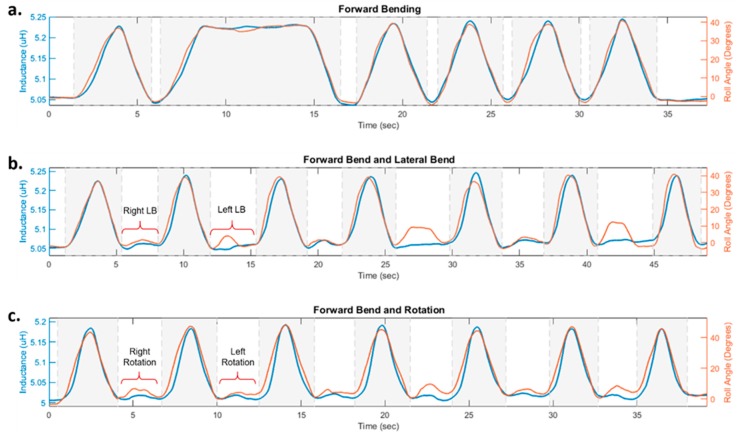
Inductance values (µH) recorded from the designed sensor and actual forward bending angles (degrees) recorded by inertial measurement units (IMUs) during the considered trunk movements: (**a**) forward bending; (**b**) forward and lateral bending; (**c**) forward bending and trunk rotation. In each case, the periods of forward bending are highlighted in grey shade.

Inductance values reported from the prototype and actual forward bending angles collected by IMUs during the testing protocol for one sample cycle of recorded movements are illustrated in Figure 7, in which the periods of forward bending are highlighted in grey shade. When the participant bent forward, the highest reported inductance value and the corresponding flexion angle were 5.245 µH and 40.911°. At the standing upright position, the inductance was measured at 5.036 µH at a flexion degree of −4.602° (Figure 7a). It is also worthwhile noting that when the bent position was held over longer durations of time, the readings of the sensor remained stable. This point is highlighted by observing Figure 7a: the second peak of the shown signal refers to a bent position held for over 10 s. The measured inductance value, i.e., the amplitude of the signal during this time is stable and around 5.225 µH, which is similar to the signal amplitude during other peaks corresponding to holding shorter bent positions (about 3 s).

Figure 7b shows results for the case in which the participant was repeating a series of forward and lateral bending movements. While the inductance values in forward bending were well above 5.1 µH, the highest inductance value in lateral bending was 5.073 µH (Figure 7b). A similar situation is observed in Figure 7c, where the inductance value during truck rotation did not go above 5.023 µH.

It is also worthwhile noting that during these tests the designed sensor demonstrated good consistency with respect to its inductance value. As observed from Figure 7, when the person was standing straight, the inductance stayed at a level of 5.050 µH. Similarly, the inductance signal maintained a level of 5.230 µH in the duration that maximum forward bent was held.

### 4.3. Comparison of Simulation and Experimental Results

To investigate how the simulation and experimental results compared, the highlights are summarized here. The inductance value of the sensor before stretching was 4.500 µH in experimental evaluation versus 4.698 µH in simulations. This difference of 4.4% might be due to small differences between the simulation and actual parameters, since the inductive textile sensor was manufactured by hand. The maximum inductance value in forward bending was expected to be 5.110 µH from simulations, while a value of 5.245 µH was obtained in experiments; indicating a small difference of 2.64% which might be due to small changes in the zigzag shape when stretched.

Since the focus of this study is on detecting forward bending movements and distinguishing them from lateral bending and twisting of the trunk, the performance of the sensor was only simulated during forward bending. Experimental evaluation of the sensor in lateral bending and twisting was carried out to better highlight how the placement of the sensor and its design and configuration result in prominent sensor response during forward bending.

### 4.4. Results of the Interference Test

**Figure 8:**
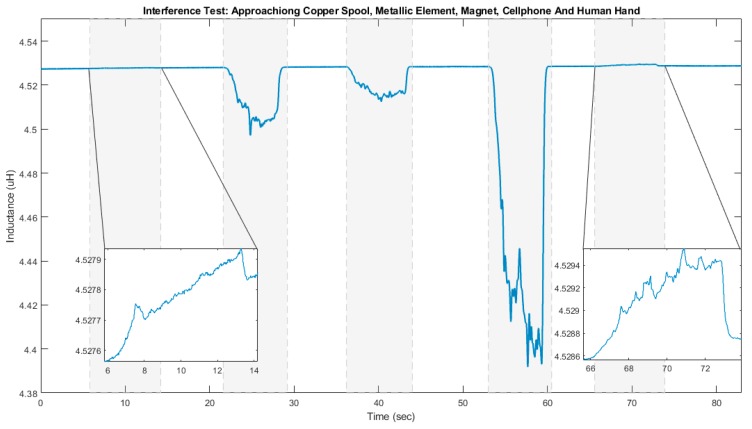
Inductance values (µH) recorded from the interference test, where a copper spool, a metallic element, a magnet, a cellphone, and a human hand were moved towards the inductive sensor’s coil. In each case, the periods of moving objects toward the coin are highlighted in grey shade.

The results of the first phase of the interference test, i.e., when the unworn prototype was extended on the table, are shown in Figure 8, in which the periods of moving different objects towards the inductive sensor’s coil are highlighted in gray shade.

The inductive sensor values were not affected by approaching the copper spool and the human hand (first and last object). More specifically, the maximum inductance change was 0.0003 µH (less than 0.01%) for the copper spool and 0.001 µH (about 0.02%) for the human hand. Approaching the metallic element and the magnet caused a maximum change of 0.030 µH (less than 1%) and 0.015 µH (less than 0.5%) in sensor readings, respectively. The object that interfered most with the inductive sensor was the cellphone with a maximum inductance change of 0.136 µH (about 3%). During this test, the cellphone was on and the Wi-Fi was activated. The cellphone case almost touched the sensor.

It is worth noting that as observed in Figure 8, the metallic element, the magnet and the cellphone decreased the inductance value, while the copper spool and the human hand increased it. In the case of the copper spool, such a result might be related to both elements (the copper spool and the inductive sensor) having the same material. The increase in the inductance value when a human hand approaches the coil might be result of a small stimulation of excitable tissues of the hand. Furthermore, the inductance changes due to the hand approaching the coil was neglectable, possibly due to the similarity between the relative magnetic permeability between biological tissues and vacuum [33]. Additionally, the inductance change between the beginning and the end of the test was 0.002 µH (about 0.04%).

**Figure 9:**
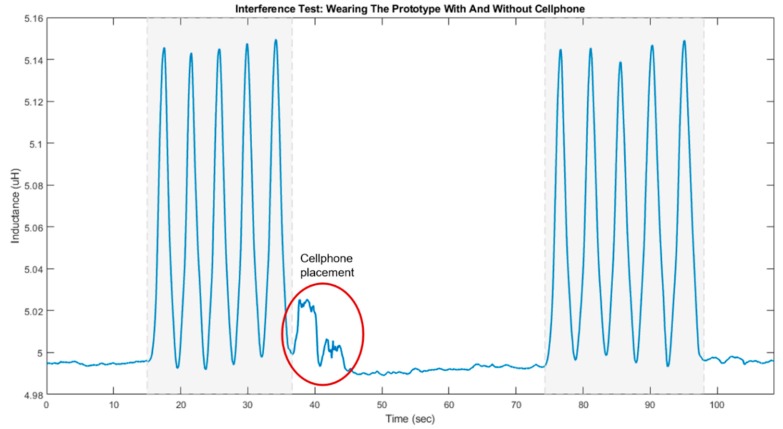
Inductance values (µH) recorded from the interference test, where a single participant was wearing the prototype and performed forward bending. In the second set of forward bend, the participant had the cellphone inside the jeans’ back pocket. In each case, the periods of forward bending are highlighted in grey shade. The red circle shows when the cellphone was put inside the back pocket.

Figure 9 illustrates the results of phase 2 of the interference test when the participant was wearing the prototype. The periods highlight in gray shade represent when the participant was bending forward. The red circle shows when the participant picked up the cellphone from the table in front and put it inside the back pocket of their jeans.

Although the cellphone caused the highest interference with sensor readings when the prototype was not worn (Figure 8), Figure 9 shows that the performance of the worn prototype was not affected: Periods of forward bending can be easily detected by observing the inductance values. Nevertheless, it should be noted that a decrease in the inductance value before and after putting the cellphone in the back pocket was observed when the user was standing upright. More specifically, the sensor reading decreased from 4.994 µH to 4.992 µH (about 0.04%).

The average change in the inductance value between standing straight and bending forward, before and after placing the cellphone in the back pocket was 0.151 µH and 0.149 µH, respectively. Therefore, the interference caused by handling the cellphone while wearing the prototype affected the signal amplitude by 1.325%. It should also be noted that although, as seen in Figure 8, moving the cellphone towards the unworn sensor noticeably decreased the inductance value, handling the cellphone while wearing the garment had a different effect. More specifically, in this case, as observed from Figure 9, moving the cellphone to the back pocket resulted in a transient increase in sensor readings. However, this increase was less than 15% of the peak inductance value corresponding to the forward bending state. Therefore, the performance of the sensor in detecting forward bends was not affected by handling the cellphone in the tested scenario.

The red circle in Figure 9 denotes the moment when the user bends forward to pick up the cellphone from the table in front and put it inside of the jean’s back pocket. It can be seen that such an action had a transient effect on sensor readings. For this test, the back pocket was chosen to recreate a more realistic scenario and also to place the cell phone in closer proximity of the sensor’s coil.

## 5. Discussion

This paper presented an inductive sensor-based wearable garment for monitoring back movements. The fabricated prototype is featured by its comfort, portability, and low power consumption that allows its operation during longer periods of time, well-suited for monitoring the back movements of users during work shifts. Textile sensors have been used in the past to monitor motion and acceleration of limbs, applied pressure and/or strain, and biosignals, such as electrocardiography (ECG) signals, electroencephalogram (EEG) signals, and respiration. Fleury et al. summarized and reported different types and applications of textile sensors in healthcare, emotion monitoring, rehabilitation, and diagnosis of sleep disorders [14]. While there are very few prototypes that fully integrate the sensing elements, wiring, and power supply into the textile, the majority of the proposed solutions implement partial integration in which only the sensing elements and wiring are embedded in the fabric [14]. The prototype presented in this paper has the inductive sensor sewn into the garment and the circuitry, including the power supply, is currently affixed to the garment using Velcro.

Table 3 provides a comparison between specifications of the prototype presented in this paper and those of similar ones in the literature that reported such measures. From Table 3, it is observed that the present prototype is lighter than its competitors. Being lightweight is an important factor for wearable devices since it directly affects the comfort of the user [24]. Power consumption is another important feature which determines the operating life of the wearable device. Table 3 shows that the prototype presented by Dionisi et al. [24] has the lowest current consumption, which might be partially due to the solar panel placed on the user’s back. However, this solution might not be effective when the user works indoors, away from sun, during long hours. The prototype presented in our study has a higher current consumption, i.e., 20.1 mA, but using a 3.7 V battery allows the device to work for more than 10 continuous hours which is sufficient for monitoring bending movements during an entire work shift.

Mattmann tested the proposed device for a larger set of movement types but reported that the device could not differentiate between similar postures and that the accuracy of detecting different postures drops from 97% to 65% when testing with a new user [29]. Tormene et al. concluded that their prototype was able to monitor forward, but not lateral, bending and proposed the placement of additional sensors [28]. Rezaei et al. proposed a wearable garment for monitoring three-dimensional movements of trunk [18]. In addition to the higher number of resistive sensors used in that prototype, the calibration was a tedious step involving implementing a machine-learning algorithm to train a model for detecting different postures [18]. The prototype presented in this paper uses a single sensor to monitor forward bending and to distinguish it from lateral bending or twisting without requiring a lengthy calibration step. It is also worth noting that while previous studies identified sliding of the clothing on the human body as one limitation that might lead to errors in detecting trunk postures [19,24], the tightfitting garment used in this study hinders sliding of the inductive sensor and helps keep the sensor in place.

The readings of the inductive textile sensor were noticeably affected when a cellphone was moved toward the unworn sensor’s coil. However, the performance of the prototype was not affected when the participant wore the prototype and performed forward bend with a cellphone inside the back pocket of the jeans the prototype. Moreover, further investigation is needed to evaluate the possible interference and performance of the prototype in the presence of implantable devices, e.g., pacemakers, defibrillators, and cochlear implants.

The prototype demonstrated good accuracy in measuring inductance, as indicated by a difference of less than 3% between its readings and those of the commercially available smart tweezers. Furthermore, obtaining the same inductance value for the same bending pose during different movements point to the high precision of the developed sensing platform. 

Unlike resistive textile sensors [29], the inductive sensor considered herein had no drift. Moreover, sensor readings had little variations, demonstrated by an average value of 5.219 µH and a standard deviation of 0.0246 µH when the bent position was held. Nevertheless, the repeatability of the sensor should be further established by additional experiments. The inductance value when the participant was standing upright was consistent during all tests. The slight differences observed in the inductance value when the participant was bending forward were mostly because the participant was not able to bend forward to the exact same bending position each time. 

The inductive textile sensor was highly sensitive to bending forward movements, while lateral bending and twisting caused small variations in sensor readings (Figure 7). This result was due to the strategic design, configuration, and placement of the sensor such that forward bending movements caused major strain on the sensor. Therefore, while previous studies had reported difficulties distinguishing between different movements while monitoring the back [28], the suggested platform can successfully indicate and report forward-bending episodes performed among other type of movements.

The zigzag pattern used in the inductive textile sensor had a significant impact on the inductance value, where without the mentioned pattern, the simulated inductance value dropped by more than 25%. Additionally, the zigzag pattern allowed embedding a non-stretchable material into a stretchable garment while preventing damage to the sensor.

The operating frequency of the sensor was calculated to be 3.46 MHz for the unworn prototype (inductance value = 4.5 µH) and 3.21 MHz for the worn prototype in maximum forward bend (inductance value = 5.245 µH). According to the Consumer and Clinical Radiation Protection Bureau, Health Canada [34], radiofrequency (RF) fields that are in the frequency range between 3 kHz and 300 GHz are safe for humans. The sensor developed in the present study has an operating frequency in the range of 3 MHz, therefore, it operates in the recommended safe limits. However, to fully establish its safety for prolonged human use, further investigation is required.

Since the focus of this study was to design a sensor to detect simple forward bending and to distinguish such movements from other movements such as twisting and lateral bending of the trunk, in the testing scenarios only simple isolated movements were considered. Moreover, in this study, the participant was asked to bend forward and stand straight at her preferred comfortable speed. Further study is required to fully characterize the behavior of the sensor during complex movements and at different movement speeds. A future prototype will also have sensors added on the waist level on both sides such that it can also detect lateral bending and trunk rotation while still discriminating between these different types of movements. Moreover, a reduction in the size of the circuitry could improve comfort by decreasing the weight of the prototype.

## 6. Conclusions

The textile inductive sensor-based wearable platform presented in this study showed excellent performance in detecting forward-bending movements, while ignoring lateral bending or trunk rotations. The designed inductive sensor had stable readings (no drift), little variations in readings during forward bends, an easy manufacturing process, and long battery life. Therefore, the proposed platform is a potential solution for preventing LBP by informing the user about the amount of strain on their lower back during long hours of work shifts. 

## Figures and Tables

**Table 1:** Parameters and zigzag characteristics used to simulate 5 single loop inductive textile sensors in Ansys. Values appearing between dashed lines indicate that the same value was used in all simulations.

Ansys’ Parameters		Sensor 1	Sensor 2	Sensor 3	Sensor 4	Sensor 5
Sensors Characteristics	Between Connections	10 mm				
	Total Height	60 mm				
	Total Length	50 mm				
	Material	Copper				
	Wire Diameter	0.14 mm				
Box Characteristics	X	100 mm				
	Y	150 mm				
	Z	100 mm				
	Material	Air				
Setup	Maximum # Passes	10				
	% Error	5				
	% Refinement Per Pass	30				
	Minimum # of Passes	5				
	Minimum Converged Passes	1				
Mesh		Classic, Small				
Excitation		1.56 mA				
Zigzag Dimensions	Width	2 mm	4 mm	6 mm	8 mm	10 mm
	Height	4.58 mm				

**Table 2:** Parameters used to simulate sensor behavior in Ansys.

Inductive Textile Sensor Simulation		
Sensor Characteristics	Distance Between Connections	15 mm
	Total Height	250 mm
	Total Length	260 mm
	Material Wire Diameter	Copper 0.14 mm
Box Characteristics	X	500 mm
	Y	450 mm
	Z	300 mm
	Material	Air
Setup	Maximum # Passes	10
	% Error	5
	% Refinement Per Pass	30
	Minimum # of Passes	5
	Minimum Converged Passes	1
Mesh	Classic, small	--
Excitation	--	1.56 mA

**Table 3:** Comparison of the present prototype against others in the literature.

Author	Type of Sensor	Integration into the Garment	Number of Sensors	Recognized Movements	Wireless	Power Consumption (mA)	Weight (g)
García Patiño, A. et al. (this paper)	Inductive	Sewn	1	Forward Bend	Yes	20.1	78.6 (Circuitry and sensor)
Rezaei, A. et al. [18]	Resistive	Sewn	18	Forward Bend Lateral Bend Rotation	No	Not specified	Not specified
Esfahani, M. I. M. et al. [19]	Resistive	Printed	12	Forward Bend Lateral Bend Rotation Mixed Movements	No	Not specified	≤ 200 (Sensors and garment)
Dionisi, A. et al. [24]	Textile Electrocardiography Electrodes (ECG) Inductive sensor (Plethysmography) 1 Accelerometer (Posture Monitoring)	Sewn (Textile Electrodes and Inductive sensor) Pocket and snap buttons (Circuit board) Not specified (Solar Panel)	2 Textile Electrodes 1 Inductive sensor 1 Accelerometer 1 Solar panel	Forward Fall Back Fall Right and Left Imbalance	Yes	9.6 (approx.)	81 approx. (solar panel and circuitry)
Tormene, P. et al. [28]	Resistive	Printed	13	Forward Bend Lateral Bend	Yes	Not specified	Not specified
Mattmann, C. [29]	Resistive	Silicone Film	21	Forward Bend Lateral Bend Rotation Lifting Shoulders Slumped Force Upright Arm Postures	Yes	Not specified	Not specified
